# Identification of Restriction-Modification Systems of *Bifidobacterium animalis* subsp. *lactis* CNCM I-2494 by SMRT Sequencing and Associated Methylome Analysis

**DOI:** 10.1371/journal.pone.0094875

**Published:** 2014-04-17

**Authors:** Mary O′Connell Motherway, Debbie Watson, Francesca Bottacini, Tyson A. Clark, Richard J. Roberts, Jonas Korlach, Peggy Garault, Christian Chervaux, Johan E. T. van Hylckama Vlieg, Tamara Smokvina, Douwe van Sinderen

**Affiliations:** 1 Alimentary Pharmabiotic Centre and School of Microbiology, National University of Ireland, Cork, Ireland; 2 Pacific Biosciences, Menlo Park, California, United States of America; 3 New England Biolabs, Ipswich, Massachusetts, United States of America; 4 Danone Nutricia Research, Palaiseau, France; Ghent University, Belgium

## Abstract

*Bifidobacterium animalis* subsp. *lactis* CNCM I-2494 is a component of a commercialized fermented dairy product for which beneficial effects on health has been studied by clinical and preclinical trials. To date little is known about the molecular mechanisms that could explain the beneficial effects that bifidobacteria impart to the host. Restriction-modification (R-M) systems have been identified as key obstacles in the genetic accessibility of bifidobacteria, and circumventing these is a prerequisite to attaining a fundamental understanding of bifidobacterial attributes, including the genes that are responsible for health-promoting properties of this clinically and industrially important group of bacteria. The complete genome sequence of *B. animalis* subsp. *lactis* CNCM I-2494 is predicted to harbour the genetic determinants for two type II R-M systems, designated BanLI and BanLII. In order to investigate the functionality and specificity of these two putative R-M systems in *B. animalis* subsp. *lactis* CNCM I-2494, we employed PacBio SMRT sequencing with associated methylome analysis. In addition, the contribution of the identified R-M systems to the genetic accessibility of this strain was assessed.

## Introduction

Bifidobacteria are a common component of the microbiota of the gastrointestinal tract (GIT) of a broad range of hosts, and their presence is associated with a positive health status of the gut [1], [2]. Compared to other micro-organisms of industrial and pharmaceutical relevance, relatively little is known about the precise molecular mechanisms that explain how bifidobacteria confer beneficial effects, although scientific progress has been made in recent times [3], [2].

A typical example of this is *Bifidobacterium animalis* subsp. *lactis* CNCM I-2494, which is a component of a fermented dairy product studied in several placebo-controlled clinical trials [4]–[6]. Veiga *et al*. [7] recently reported on the marked reduction in intestinal inflammation observed in a murine model of inflammatory bowel disease (IBD) upon administration of fermented milk containing *B. animalis* subsp. *lactis* CNCM I-2494, while Agostini et al [8] demonstrated that a dairy product containing this strain reduces visceral hypersensitivity associated with acute stress in a murine model. However, the molecular mechanisms of these observations remain unclear.

Insights into bacterial modes of action may be obtained by comparative and functional genomic approaches. The last decade has seen a vast expansion of complete genome sequences obtained from various human, animal or insect-derived bifidobacterial species [9]. While these have contributed very significantly to advancing our knowledge on bifidobacterial genomics, genetics and metabolism, the availability of a genome sequence is merely a first step towards a better understanding of the beneficial attributes of a specific strain. The complete genome sequence of CNCM I-2494 has been reported [10] and genetic determinants potentially involved in host interaction have been identified. However, demonstration of the role and functionality of these determinants demands the availability of suitable molecular tools. In this respect, the genus *Bifidobacterium* is renowned for being genetically recalcitrant and while numerous *E. coli*-bifidobacterial shuttle vectors have been created [11], the successful application of these vectors can be strain-dependent. At present the ability to access the chromosome to create mutants via site-specific homologous recombination approaches remains feasible for relatively few bifidobacterial strains [12], [13], while transposon mutagenesis has only very recently been described [14]. Merely 5 years ago we published the first method for the creation of site-specific homologous recombination mutants in *Bifidobacterium breve* UCC2003 by adopting a well-known lactococcal system [15], although this method was impractical for routine laboratory use [16]. The intractable genetic characteristics of bifidobacteria are attributed, at least in part, to restriction-modification (R-M) systems harbored by each bifidobacterial species or strain [17]–[19]. We have exploited this knowledge for our prototype *Bifidobacterium* strain, *B. breve* UCC2003, and have been successful in creating insertion mutants via site-specific homologous recombination [17]. The ability to create isogenic mutants has advanced our understanding of *B. breve* UCC2003 strain colonization and adaptation factors [20]–[30]. While insertion mutants have been created in other strains of *B. breve* [25],[31], and representative strains of other bifidobacterial species [32]–[34], the variability in transformation efficiency observed between strains does not allow mutant construction in all bifidobacteria. *B. animalis* subsp. *lactis* CNCM I-2494 is of particular importance in the composition of a fermented dairy product that has been commercialized since 1987. The low transformation efficiency of this strain has prevented the development of genetic tools needed to facilitate the molecular characterization of the beneficial effects associated with this product.

In this study we have resequenced the genome of *B. animalis* subsp. *lactis* CNCM I-2494 adopting PacBio's single molecule real-time (SMRT) sequencing coupled to methylome analysis [35], [36] to unveil the strain-specific complement of methylated recognition sequences within the genome. Through a combination of methods we deduced the methylase-associated recognition sequence. Furthermore, we determined the contribution of each R-M system to the genetic accessibility of *B. animalis* subsp. *lactis* CNCM I-2494.

## Experimental Procedures

### Bacterial strains, plasmids and culture conditions

Bacterial strains, bacteriophage and plasmids used in this study are detailed in Table 1. *B. animalis* subsp. *lactis* CNCM I-2494 was routinely cultured in modified-MRS [37], designated here as mMRS, prepared from first principles [peptone from casein, 10 g L^−1^; meat extract, 10 g L^−1^ and yeast extract, 5 g L^−1^ (purchased from Difco); K_2_HPO_4_, 3 g L^−1^; KH_2_PO_4_, 3 g L^−1^; pyruvic acid, 0.2 g L^−1^; polysorbate 80, 1 ml L^−1^; tri-ammonium citrate, 2 g L^−1^; MgSO_4_.7H_2_O, 0.575 g L^−1^; MnSO_4_.4H_2_O, 0.12 g L^−1^; cysteine-HCl, 0.3 g L^−1^; and FeSO_4_.7H_2_O, 0.034 g L^−1^], and supplemented with 0.05% cysteine-HCl and 1% maltose prior to inoculation. *B. animalis* subsp. *lactis* CNCM I-2494 (or plasmid-containing derivatives) were cultivated at 4°C under anaerobic conditions which were maintained using an Anaerocult oxygen depleting system (Merck, Darmstadt, Germany) in an anaerobic chamber. *Escherichia coli* strains were cultured in Luria-Bertani broth (LB) [38] at 37°C with agitation, while *L. lactis* was grown in M17 broth (Oxoid, UK) supplemented with 0.5% glucose at 30°C. Where appropriate, growth media contained ampicillin (Amp; 100 µg ml^−1^ for *E. coli*), chloramphenicol (Cm; 5 µg ml^−1^ for *L. lactis*), tetracycline (Tet; 10 µg ml^−1^ for *E. coli*), kanamycin (kn; 50 µg ml^−1^ for *E. coli*) or spectinomycin (Spec; 100 µg ml^−1^ for *E. coli* or bifidobacteria). Unless otherwise specified all media components and antibiotics were purchased from Sigma Aldrich (Wicklow, Ireland)

**Table 1:** Bacterial strains, bacteriophage and plasmids used in this study.

Strain or plasmid	Relevant characteristics	Reference or Source
**Strains**		
*B. animalis* subsp. *lactis* CNCM I-2494		Danone
		
*E. coli strains*		
EC101	Cloning host, km^r^	[15]
Xl1Blue	Cloning host, tet^r^	Stratagene
MG1655	Host for plaque assays	(62)
EC100	Host for plaque assays	EPICENTRE Biotechnologies, Inc.
*Lactococcus lactis* NZ9000	MG1363, *pep*N::*nis*RK, nisin-inducible overexpression host	[63]
**Bacteriophage**		
P1*vir*-MG1655	P1*vir* phage propagated on *E. coli* MG1655 for plaque assays	UCC, School of Microbiology bacteriophage collection
P1*vir*-EC100	P1*vir* phage propagated on *E. coli* EC100 for plaque assays	UCC, School of Microbiology bacteriophage collection
**Plasmids**		
pAM5	pBC1-puC19-Tc^r^	[45]
pDG7	pMB1-Cm^r^-Amp^r^	[46]
pMG36S	Spectinomycin resistance plasmid (Spec^r^)	Molgen plasmid collection, Groningen, The Netherlands
pDM1	pAM5 derivative containing spectinomycin resistance casette	This study
pDM2	pDG7 derivative containing spectinomycin resistance casette	This study
pNZ44	Cm^r^, expression vector	[47]
pWSK29	Amp^r^, low copy number *E. coli* cloning plasmid	[48]
pWSK29-M.BanLI	pWSK29 derivative containing *M.banLI*	This study
pWSK29-M.BanLII	pWSK29 derivative containing *M.banLII*	This study
pWSK29-RM.BanLI	pWSK29 derivative containing *RM.banLI*	This study
pWSK29-M.BanLI- M.BanLII	pWSK29 derivative containing *M.banLI* and *M.banlII*.	This study

### Nucleotide sequence analysis

Sequence data were obtained from the Artemis-mediated [39] genome annotations of the *B. animalis* subsp. *lactis* CNCM I-2494 [10]. Database searches were performed using non-redundant sequences accessible at the National Centre for Biotechnology Information internet site (http://www.ncbi.nlm.nih.gov) using BLAST [40], [41] and the REBASE [42] site accessible at the New England Biolabs internet site (www.neb.com/rebase). Sequence assembly, verification and analysis were performed using the Seqman and Seqbuilder programs of the DNASTAR software package (DNASTAR, Madison, WI, USA).

### DNA manipulations

Chromosomal DNA was isolated from bifidobacteria as previously described [43]. Minipreparation of plasmid DNA from *E. coli* or *B. animalis* subsp. *lactis* CNCM I-2494 was achieved using the Qiaprep spin plasmid miniprep kit (Qiagen GmBH, Hilden, Germany). For bifidobacteria an initial lysis step was incorporated into the plasmid isolation procedure, cells were resuspended in lysis buffer supplemented with lysozyme (30 mg ml^−1^) and incubated at 37°C for 30 min. Procedures for DNA manipulations were performed essentially as described by Sambrook *et al*. [38]. Restriction enzymes, shrimp alkaline phosphatase and T4 DNA ligase were used according to the supplier's instructions (Roche Diagnostics, Bell Lane, East Sussex, UK). Synthetic single stranded oligonucleotide primers used in this study were synthesized by MWG Biotech AG (Ebersberg, Germany). Standard PCRs were performed using TaqPCR mastermix (Qiagen), while high fidelity PCR was achieved using PfuII Ultra polymerase (Agilent, Limerick, Ireland). PCR fragments were purified using the Qiagen PCR purification kit (Qiagen). Electroporation of plasmid DNA into *E. coli* was performed as described by Sambrook *et al*. [38], and into *L. lactis* as described by Wells *et al*. [44]. The correct orientation and integrity of all constructs was verified by DNA sequencing, performed at MWG Biotech (Ebersberg, Germany).

### PacBio SMRT sequencing and methylome analysis

DNA isolation, purification, SMRT bell library preparation were performed as previously described [35], [36]. SMRT sequencing was performed on a PacBio *RS* instrument (Pacific Biosciences, Menlo Park CA, USA). Sequencing reads were processed and mapped to the sequence of *B. animalis* subsp. *lactis* CNCM I-2494 (Genbank accession number CP002915). Interpulse durations were measured as previously described [35]. To identify methylated positions the Pacific Biosciences SMRTPortal analysis platform (version 1.4) was adopted, this employs an *in silico* kinetic reference and a t-test based kinetic score detection of modified base positions.

### Construction of the *E.coli*-bifidobacterial shuttle vectors pDM1 and pDM2

The spectinomycin resistance gene, including its presumed promoter region, was amplified from pMG36S using primer combinations SpecF and SpecR, which harbor SacI sites at their 5′ end, or SpecF1 and SpecR1, which contain EcoR1 and HindIII sites, respectively. In each case the 1117-bp amplicon was digested with either SacI, or a combination of EcoRI and HindIII, and ligated to similarly digested pAM5 [45] or pDG7 [46], respectively. The ligations were transformed into *E. coli* EC101 with selection on LB agar containing kanamycin and spectinomycin. A number of transformants were selected and screened for plasmid content by restriction analysis and DNA sequencing. In one of the recombinant plasmids, which was termed pDM1, the tet^r^ cassette of pAM5 had been replaced with the spectinomycin resistance cassette, while in the second recombinant plasmid, designated pDM2, the spectinomycin cassette had been cloned in the unique EcoRI and HindIII sites of pDG7.

### Transformation of *B. animalis* subsp. *lactis* CNCM I-2494

48 ml of mMRS including the supplemented (0.05%) cysteine (1%) maltose was inoculated with 2 ml of an overnight culture of *B. animalis* subsp. *lactis* CNCM I-2494 and incubated anaerobically at 42°C. At an optical density (OD_600 nm_) of approximately 0.8, bacterial cells were collected by centrifugation at 4,700 × g for 10 min at 4°C, and the resulting cell pellet was then washed twice with chilled sucrose citrate buffer (1 mM citrate [pH 5.8], 0.5 M sucrose). The cells were subsequently suspended in 300 µl of chilled sucrose citrate buffer. Fifty microliters of the cell suspension was used for each electrotransformation, and cells and plasmid DNA were mixed and held on ice prior to the pulse at 25 µF, 200 Ohms and 2.5 kV. After transformation, cells were suspended in 1 ml of mMRS supplemented with cysteine and maltose, and incubated for 3 hours at 42°C. Serial dilutions were plated on RCA supplemented with maltose and containing the appropriate antibiotic and incubated at 42°C for 24–36 h to allow visible colonies to form.

### Cloning of the BanLI R-M system and bacteriophage plaque assays

For the construction of plasmid pWSK29-RM.BanLI, DNA fragments encompassing the coding sequences of *banLI.RM* (corresponding to locus tags BALAC2494_1402 and BALAC2494_1401) were generated by PCR amplification employing chromosomal DNA of *B. animalis* subsp. *lactis* CNCM I-2494 as a template, and using PFU Ultra DNA polymerase plus primer combinations RMBanLIF and RMBanLIR (Table S1). The forward primer contained the sequence of a ribosome binding site to facilitate translation of corresponding mRNA following transcriptional fusion to the *lac* promoter on pWSK29. The amplified fragment was restricted with NotI and PstI, and ligated to similarly digested pWSK29. The ligation was then used to transform *E. coli* Xl1Blue. The plasmid content of a number of Amp^r^ transformants was screened by restriction analysis and the integrity of positively identified clones was verified by sequencing, and one of the thus identified plasmids was designated pWSK29-RM.BanLI. This construct was transferred to the phage sensitive host *E. coli* DSMZ5911 by electroporation to facilitate plaque assays. Plaque assays were performed in accordance with standard procedures [38] adopting serial dilutions of two P1*vir* phage lysates generated following propagation of P1*vir* on *E. coli* strains MG1655 or EC100. Agar plates were incubated at 37°C for 24 hours after which the number of plaque forming units for each phage and host combination was enumerated.

### Cloning of genes encoding putative methyltransferases

For construction of plasmids pWSK29-M.BanLI and pWSK29-M.BanLII, DNA fragments encompassing *banLI.M* (BALAC2494_1402) and *banLII.M* (BALAC2494_0032) were generated by PCR amplification employing chromosomal DNA of *B. animalis* subsp. *lactis* CNCM I-2494 as a template, and using PFU Ultra DNA polymerase plus primer combinations BanLIF and BanLIR, or BanLIIF and BanLIIR, respectively (Table S1). Each forward primer contained the sequence of a ribosome binding site to facilitate translation of corresponding mRNA. For the *banLI.M*-encompassing fragment PstI and HindIII sites were incorporated into the forward and reverse primers, respectively, to facilitate ligation to similarly digested pNZ44 [47]. Ligations were introduced into *L. lactis* NZ9000 by electroporation and transformants were selected on chloramphenicol resistance. The presence and integrity of the cloned insert in one of the recombinant plasmids, designated pNZ44-BanLI, was confirmed by restriction and sequence analysis. The region encompassing the *banLI.M* coding sequence and the constitutive p44 lactococcal promoter, specified by pNZ44, was amplified by PCR from a representative pNZ44-BanLI plasmid using primer pair BanLIF1 and BanLIR. The resultant fragment was restricted with BglII and HindIII and ligated to BamHI and HindIII-restricted pWSK29 [48]. The ligation mixture was introduced into *E. coli* Xl1Blue by electroporation and several of the resulting Amp^r^ transformants were then checked for plasmid content and, where relevant, sequence integrity.

Transcriptional fusion of the gene to the *lac* promoter on pWSK29 was achieved by cloning a *banLII.M*-encompassing amplicon, which had been restricted with XbaI and PstI (sites included in the primers), into similarly digested pWSK29. Each ligation was transformed into *E. coli* Xl1Blue. Plasmid content of a number of Amp^r^ transformants was screened by restriction analysis. The integrity of positively identified plasmids was verified by sequencing, of which one was designated pWSK29-M.BanLII.

To construct plasmid pWSK29-M.BanLI-M.BanLII, which expresses both of the identified *B. animalis* subsp. *lactis* CNCM I-2494 methylases, the DNA fragment encompassing p44-M.BanLI was amplified from pWSK29-M.BanLI by PCR using primer combinations BanLIF2 and BanLIR2 (Table S1), which had SalI restriction sites incorporated at their 5′ ends. The resulting 1347 bp amplicon was digested with SalI, ligated into similarly digested pWSK29-M.BanLII, followed by introduction into *E. coli* Xl1Blue by electrotransformation and selection on ampicillin resistance. Plasmid content of a number of Amp^r^ transformants was screened by restriction analysis and the expected sequence integrity of positively identified clones was verified by sequencing.

## Results

### Genetic organization and amino acid analysis of the BanLI, BanLII and BanLIII R-M systems from *B. animalis* subsp. *lactis* CNCM I-2494

Three loci, predicted to encode two apparently complete R-M systems, designated BanLI and BanLII, and a single incomplete R-M system, termed BanLIII and represented by an apparent orphan methylase, were identified on the genome sequence of *B. animalis* subsp. *lactis* CNCM I-2494. The G+C content of the genes encoding each of the complete R-M systems is 50%, which is lower than the overall G+C content for bifidobacteria (60%) and also lower than the G+C content for this strain (60.49%), while the G+C content of the gene specifying the predicted orphan methylase is 67.88%, higher than that of the genome. The first gene of the BanLI system (BALAC2494_1403) encodes a protein that shows homology to putative phage integrases from *B. animalis* subsp. *lactis* and *B. longum* subsp. *infantis* ATCC15697. The second gene, designated *banLI.M*, encodes a protein (M.BanLI; 41.68 kDa) with homology to adenosine-specific methyltransferases. This gene is conserved in all available genome sequences of *B. animalis* subsp. *lactis* (Fig. S1). In addition, the protein product shares 62% and 53% identity with predicted methyltransferases from *B. bifidum* strains LMG13195 and NCIMB41171, respectively. Two of the four conserved motifs characteristic of the N12 class of N6-adenosine-methyltransferase, CMI and CMII [49] can be identified in M.BanLI (Fig. S2A). The third gene of the BanLI gene cluster, *banLI.R*, is conserved in all *B. animalis* subsp. *lactis* genome sequences, and encodes a 43.5 kDa protein, which exhibits similarity to various type II R-M system restriction subunits and for this reason is presumed to represent the restriction component of the BanLI R-M system.

The second identified R-M system encoded by the genome of *B. animalis* subsp. *lactis* CNCM I-2494 (and other sequenced *B. animalis* subsp. *lactis* strains), BanLII, shares 52% identity with predicted R-M system methylases encoded by *B. dentium* strains Bd1 and ATCC27678. The BanLII R-M system is specified by two genes with a convergent orientation and is predicted to represent an isoschizomer of AvaII. The first gene, *banLII.M*, encodes a 502 amino acid protein (56.68 kDa), which shares 42% identity with M.AvaII, a 5′-methylcytosine methyltransferases [50], [51]. Consistent with this designation, the six highly conserved motifs of cytosine-specific MTases are all present in M.BanLII (Fig. S2B). The second gene *banLII.R* encodes a 317 amino acid protein (36.21 kDa), and, based on its location and corresponding GC content, is predicted to encode a restriction endonuclease, although no significant similarity to any known restriction endonucleases was observed on performing rebase searches.

The third RM system, designated BanLIII is predicted not to be functional due to the first gene of the cluster, represented by BALAC2494_259, being a suspected pseudogene. The protein remnant encoded by BALAC2494_259 shows similarity to the helicase domain of the Restriction-subunit of Type III R-M systems. The methyltransferase (191 amino acids; 20.75 KDa) predicted to be encoded by BALAC2494_260, and designated MBanLIII, shows 61% identity to predicted adenosine-methyltransferases from *B. bifidum* strains NCIMB41171 and BGN4. Interestingly, this gene appears to be absent in the majority of *B. animalis* subsp. *lactis* genome sequences, being only identifiable in the genome of *B. animalis* subsp. *lactis* ADO11, while a homolog with 94% identity was present on the genome of *B. animalis* subsp. *animalis* ATCC25527.

### PacBio SMRT Genome sequencing and Methylome analysis

**Figure 1:**
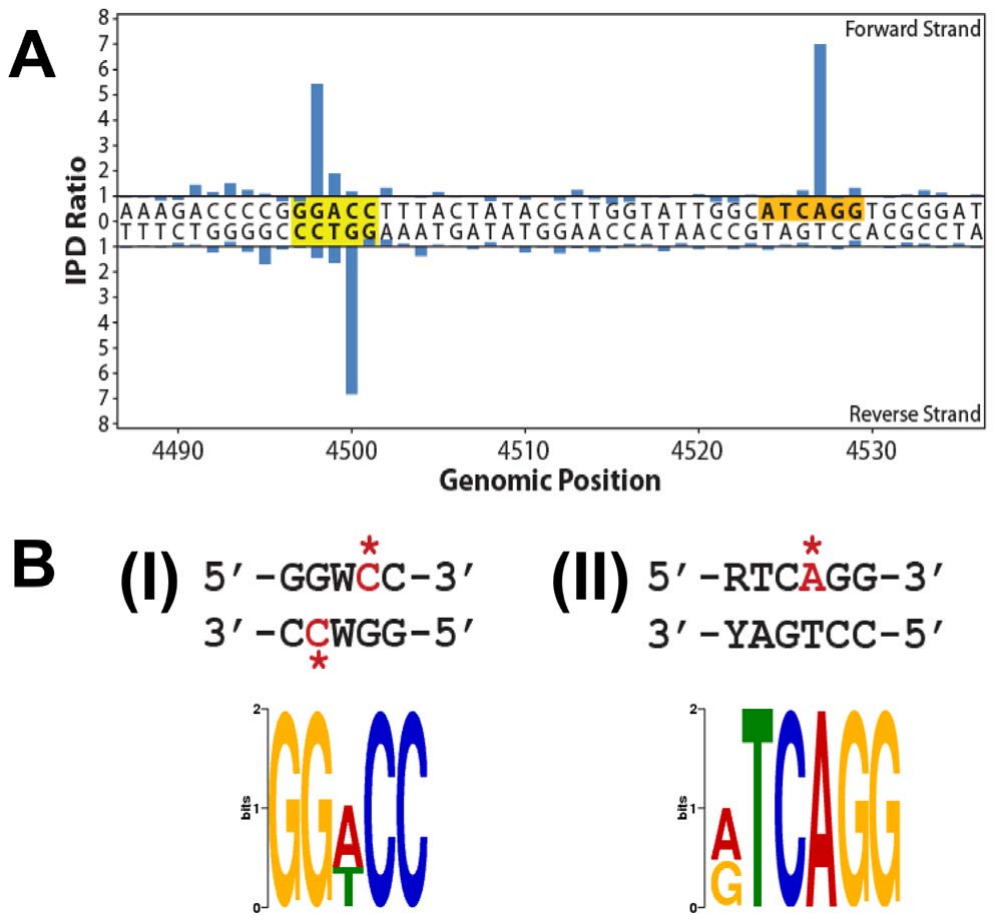
Methylome determination for *B. animalis* subsp. *lactis* CNCM I-2494. Methylated motifs from the methylome analysis are depicted in Panel A. Methylated motifs are highlighted in yellow (5mC) or orange (6mA). Panel B, (I) and (II) illustrate the MTase specificities determined from the genomic positions detected as methylated. The methylated positions are indicated by red text with an asterisk.

In order to identify methylated bases on the genome of *B. animalis* subsp. *lactis* CNCM I-2494 [10] and to determine if the identified methylases were responsible for such methylation, the genome of this strain was subjected to SMRT sequencing coupled to a subsequent methylome analysis. Two SMRTbell libraries of power-cleaned genomic DNA were prepared, five SMRT Cells were run using a 10 kb library and adopting the XL polymerase enzyme during which a 120 minute movie was captured. Using this approach the kinetic signature of 5-mC was shown to be weak, making detection during SMRT sequencing challenging. For this reason a conversion of 5-mC to 5-caC (5-carboxyl cytosine) using the Tet1 enzyme (WiseGene), as a means of enhancing the detection of 5-mC, was performed on genomic DNA randomly sheared to 1.5 kb and subsequently converted into a SMRTbell library [52], [53]. Four SMRT cells were run using the C2 polymerase enzyme and two 45 minute movies were captured. For each library the methylome was determined using SMRT Analysis software version 1.4. Genome wide motif analysis resulted in the identification of 5′-RTC^6 m^AGG-3′ and 5′-GGW^5 m^CC-3′ as the 6 mA and 5 mC recognition sequences, respectively (Fig. 1). The extent of methylation across the genome at 5′-RTC^6 m^AGG-3′ was expected to be 1576 positions, all of which were indeed detected by the methylome analysis, while for 5′-GGW^5 m^CC-3, the AvaII recognition sequence, 4420 positions were present on the genome, of which 4124 (93.3%) were detected as being methylated based on our methylome analysis.

### Assessment of restriction activity in *B. animalis* subsp. *lactis* CNCM I-2494

**Figure 2:**
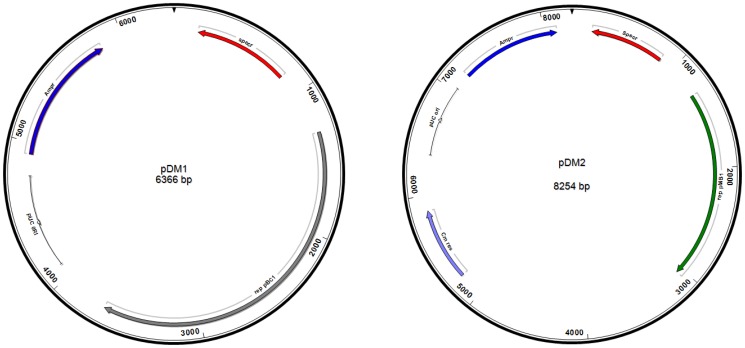
Schematic representation of the *E. coli*-bifidobacterial shuttle vectors pDM1 and pDM2. pDM1 is a derivative of pAM5 where the tetracycline resistance gene has been replaced with the spectinomycin resistance cassette. pDM2 is a derivative of the plasmid pDG7 where the spectinomycin resistance cassette was cloned in the unique EcoR1 and HindIII sites of pDG7.

**Figure 3:**
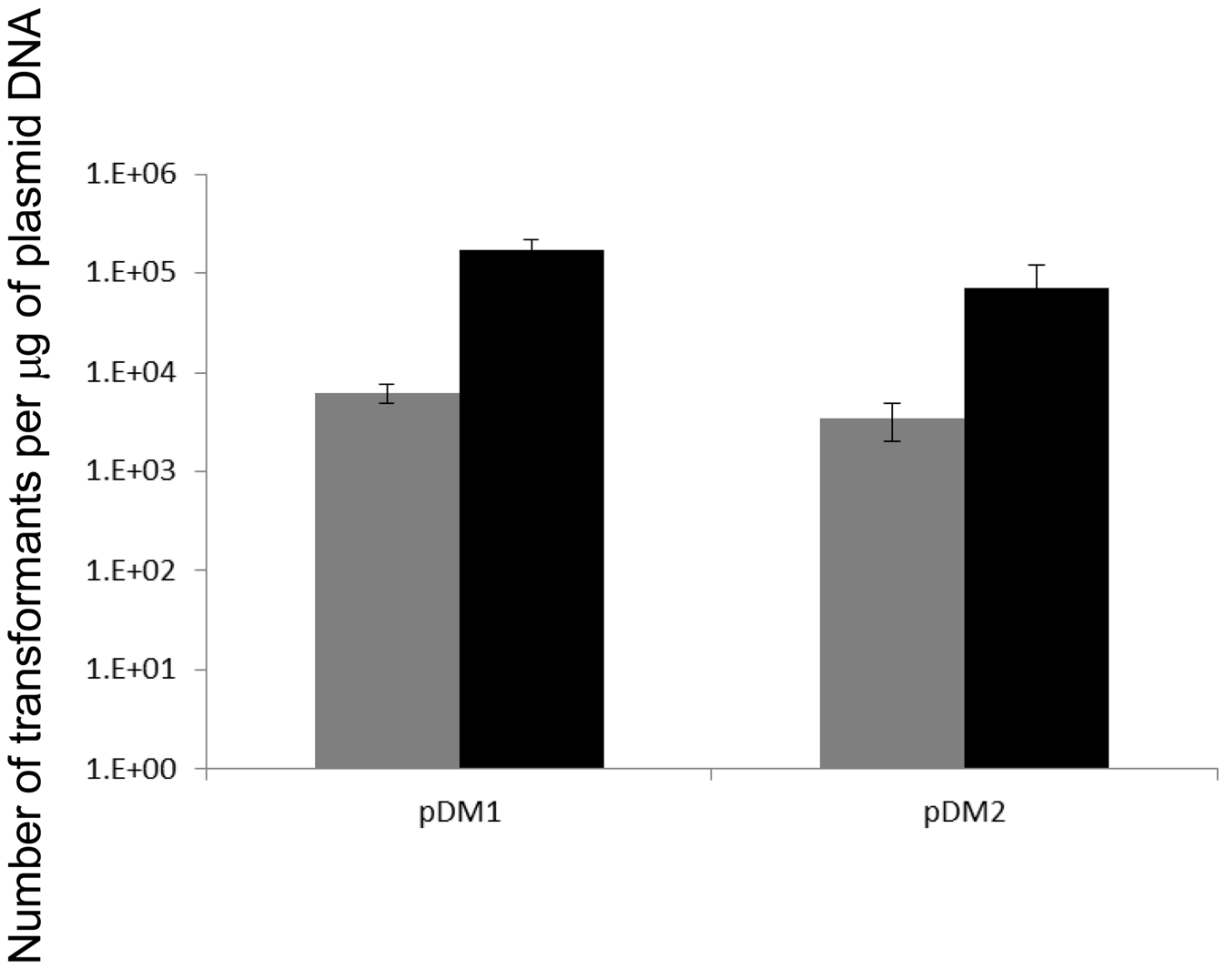
Transformation of *B. animalis* subsp. *lactis* CNCM I-2494. pDM1 or pDM2 DNA was isolated from *E. coli* (grey bars) or *B. animalis* subsp. *lactis* CNCM I-2494 (black bars). Data presented are averages of triplicate experiments.

To establish if the identified R-M-systems are functional in *B. animalis* subsp. *lactis* CNCM I-2494 and whether or not they affect plasmid-mediated transformation efficiency of this strain, the transformation frequency of two *E. coli*-bifidobacterial shuttle vectors, pDM1 and pDM2 (Fig. 2), was determined when these plasmids had been isolated either from *B. animalis* subsp. *lactis* CNCM I-2494 (in which case the plasmid DNA is assumed to be protected from R-M) or from *E. coli* (such DNA would be expected to be sensitive to the bifidobacterial R-M systems). 200 ng quantities of each of these plasmid DNA preparations, isolated from these two different hosts, were used to transform *B. animalis* subsp. *lactis* CNCM I-2494 by electroporation. Transformants were selected on RCA supplemented with 1% maltose and spectinomycin, and enumerated following anaerobic incubation at 42°C for 36 hours. There was an approximately 27 and 20 fold higher transformation efficiency for the pDM1 and pDM2 plasmid DNAs, respectively, isolated from *B. animalis* subsp. *lactis* CNCM I-2494 as compared to corresponding plasmid DNA isolated from *E. coli* (Fig. 3), thus clearly indicating that one or more of the identified R-M systems encoded by *B. animalis* subsp. *lactis* CNCM I-2494 is functional and affects the efficiency at which plasmids can be introduced in this strain.

### M.BanLI and M.BanLII represent two functional R-M methyltransferases in *B. animalis* subsp. *lactis* CNCM I-2494

To determine the precise MTase activity of M.BanLI, the coding sequence of *banLI.M* together with a sequence specifying a synthetic ribosome binding site (ATAAGGAGGCACTCACC) were firstly cloned downstream of the p44 promoter on pNZ44, to generate pNZ44-banLI.M. This *banLI.M*-encompassing sequence together with the p44 promoter was subsequently amplified from pNZ44-banLI.M by PCR and cloned in pWSK29 to generate pWSK29-M.BanLI (see Experimental Procedures for details). Plasmid DNA preparations of pWSK29 (control) or pWSK29-M.BanLI, each isolated from *E.coli* XL1Blue, were analysed by PacBIO SMRT sequencing and subsequent methylome analysis. The native R-M systems in *E. coli* XL1Blue, M.EcoKDam and M.EcoKI, methylated the plasmid complement of pWSK29 (control) or pWSK29-M.BanLI at 5′-G^6 m^ATC-3′ and 5′-A^6 m^ACNNNNNNGTGC-3′, respectively (data not shown). An additional single stranded 6 methyl adenine sequence, 5′-RTC^6 m^AGG-3′, was detected at nine positions across the sequence of pWSK29-M.BanLI, thereby attributing M.BanLI to this novel methylation activity encoded by *B. animalis* subsp. *lactis* CNCM I-2494.

**Figure 4:**
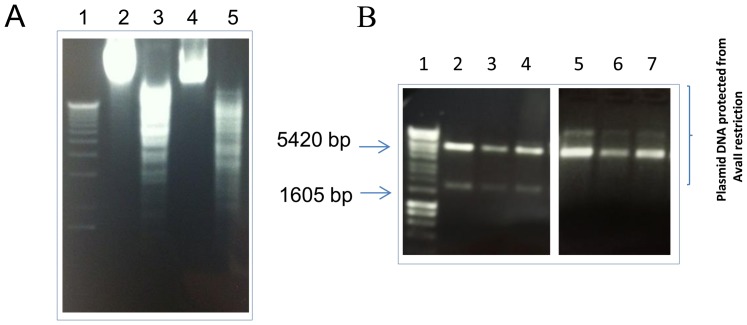
**(A)**. Restriction analysis of total DNA from *B. animalis* subsp. *lactis* CNCM I-2494. Lane1, molecular weight marker X (Roche). Lane 2: Unrestricted total DNA from *B. animalis* subsp. *lactis* CNCM I-2494, Lane 3–5 total *B. animalis* subsp. *lactis* CNCM I-2494 DNA restricted with lane 3, HindIII; lane 4, AvaII and lane 5, EcoRI. **(B)** Restriction analysis of plasmid DNA isolated from three representative *E. coli* Xl1Blue -pWSK29-M.BanLII. Plasmid DNA from *E. coli* Xl1Blue -pWSK29-M.BanLII restricted with PstI and XbaI, lanes 2–4 (expected and obtained, product sizes of PstI +XbaI digests are 5420 bp and 1605 bp), or AvaII, lanes 5–7.

In order to verify the prediction that *B. animalis* subsp. *lactis* also encodes a distinct MTase that protects, based on sequence similarity to characterised R-Ms and on the obtained methylome data, DNA sequences restricted by AvaII, genomic DNA of *B. animalis* subsp. *lactis* CNCM I-2494 was restricted by AvaII and analysed by agarose gel electrophoresis. The results obtained indeed demonstrated that the *B. animalis* subsp. *lactis* CNCM I-2494 genomic DNA is protected from restriction with AvaII (Fig. 4A), thereby suggesting that the predicted cytosine methyltransferase M.BanLII is responsible for methylation at this recognition sequence. To verify this prediction we constructed pWSK29-M.BanLII, in which the coding sequence of *banLII.M* was transcriptionally fused to the *lac* promoter on pWSK29. Plasmid DNA preparations of pWSK29-M.BanLII isolated from *E.coli* XL1Blue, were indeed insensitive to digestion with AvaII (Fig. 4B). Collectively, these results demonstrate that the genomic methylation patterns of the *B. animalis* subsp. *lactis* CNCM I-2494 can be attributed to two R-M methyltransferases, namely M.BanLI and M.BanLII, that methylate 5′-RTC^6 m^AGG-3′ and 5′-GGW^5 m^CC-3′, respectively and that can be successfully expressed in the heterologous host *E. coli*.

### Expression of M.BanLI and M.BanLII in *E. coli* and methylation of plasmid DNA

In order to assess the impact of each identified methylase on the transformation efficiency of *B. animalis* subsp. *lactis* CNCM I-2494 by prior methylation of plasmid DNA, we adopted *E. coli* XL1Blue-pWSK29-M.BanLI and *E. coli* XL1Blue-pWSK29-M.BanLII (expressing M.BanLI and M.BanLII, respectively, and as described above). To evaluate the effect of methylation of plasmid DNA on transformation efficiency, pDM1 was introduced into *E. coli* XL1blue that already harbored pWSK29-M.BanLI or pWSK29-M.BanLII. Plasmid preparations of *E. coli* pWSK29-M.BanLI + pDM1 or *E. coli* pWSK29-M.BanLII + pDM1 were then used for *B. animalis* subsp. *lactis* CNCM I-2494 transformation experiments. pDM1 DNA isolated from *E. coli* harboring pWSK29-M.BanLI gave approximately a 1.8-fold higher transformation frequency as compared to pDM1 from *E. coli* pWSK29, while plasmid DNA isolated from *E. coli* pWSK29-M.BanLII gave a 16-fold higher transformation frequency, thereby demonstrating that each individual R-M system does contribute to a different and in one case very modest degree to the genetic accessibility of *B. animalis* subsp. *lactis* CNCM I-2494.

The above data also revealed a disparity between the transformation results and the number of R.BanLI and R.BanLII recognition sites on pDM1. pDM1 harbours 13 R.BanLI recognition sequences (5′-RTCAGG-3′) and 3 R.BanLII recognition sequences (5′-GGWCC-3′), and despite this a higher transformation efficiency was observed upon methylation of pDM1 with M.BanLII. To address the unexpectedly low impact of M.BanLI-mediated pDM1 methylation on transformation efficiency and to establish if R.BanLI was indeed active, the complete BanLI R-M system was cloned downstream of the *lac* promoter on pWSK29 (see experimental procedures) to generate pWSK29-RMBanLI. This construct was introduced into *E. coli* DSMZ5911 to generate *E. coli* DSMZ5911-pWSK29-RMBanLI. This strain and *E. coli* DSMZ5911 or *E. coli* DSMZ5911-pWSK29 (controls) were used for plaque assays with *E. coli* P1*vir* bacteriophage,that was propagated on *E. coli* MG1655 or *E. coli* EC100. The efficiency of plaquing (EOP) for each phage preparation on *E. coli* DSMZ5911-pWSK29-RMBanLI relative to the control strains (EOP  = 1) was approximately 10^-1^ for each phage, thereby demonstrating that R.BanLI is active but apparently has very limited restriction activity under the conditions used (Fig. S3).

**Figure 5:**
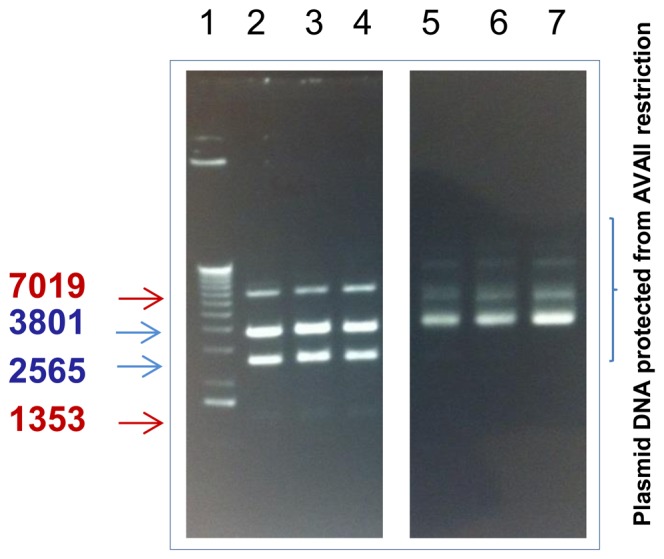
Restriction analysis of plasmid DNA. Restriction analysis of total plasmid DNA isolated from three representative *E. coli* Xl1Blue-pWSK29-M.BanLI-M.BanLII transformants harbouring pDM1. Lane 1, molecular weight marker X (Roche). Total plasmid DNA from *E. coli* Xl1Blue -pWSK29-M.BanLI-M.BanLII + pDM1 restricted with Sal1, lanes 2–4 or AvaII, lanes 5–7. The sizes of the expected and obtained SalI restriction fragments for pWSK29-M.BanLI-M.BanLII component of the total plasmid preparation are 7019bp and 1353bp (and indicated in red) while for pDM1 the expected restriction fragment sizes are 3801bp and 2565bp (and indicated in blue). The expected SalI digest restriction fragment sizes for the total plasmid complement of *E. coli* Xl1Blue -pWSK29-M.BanLI-M.BanLII + pDM1 are indicated to the left of the figure.

**Figure 6:**
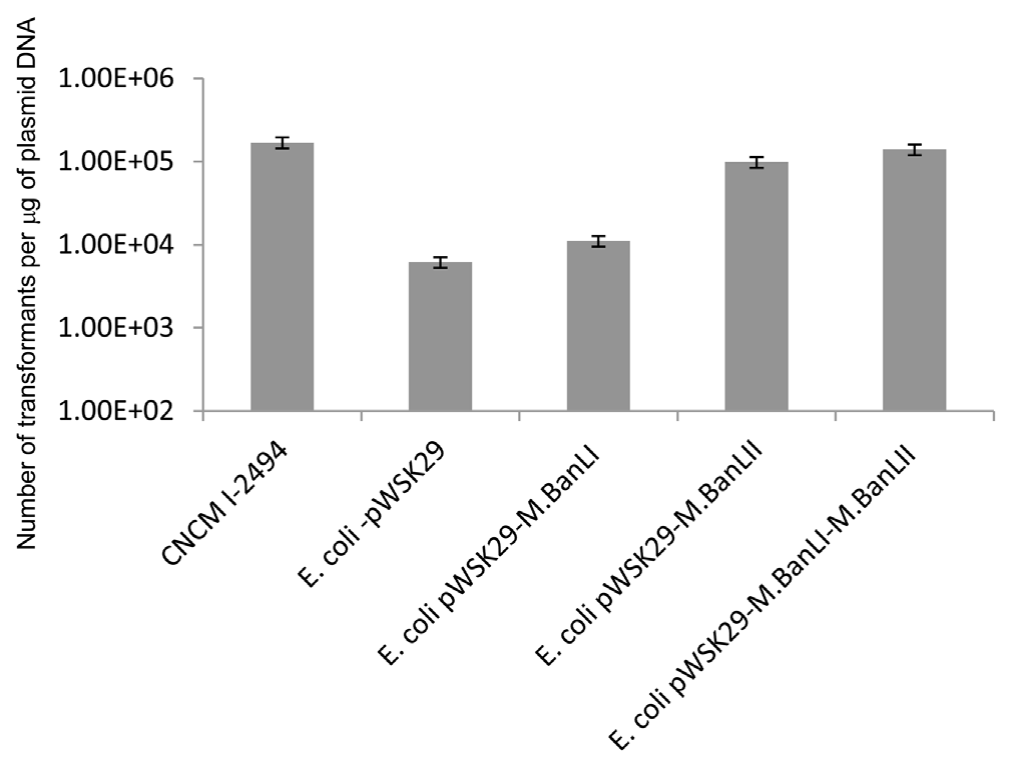
Transformation efficiency of *B. animalis subsp. lactis* CNCM I-2494. Transformation with pDM1 plasmid DNA isolated from CNCM I-2494, *E. coli* pWSK29, *E. coli* pWSK29-M.BanLI, *E. coli* pWSK29-M.BanLII or *E. coli* pWSK29-M.BanLI-M.BanLII. Data presented are averages of triplicate experiments.

In order to examine if the combined methylation of pDM1 by M.BanLI and M.BanLII would further increase the transformation efficiency of *B. animalis* subsp. *lactis* CNCM I-2494 an additional *E. coli* strain harbouring pWSK29-M.BanLI-M.BanLII was constructed (see Experimental Procedures). pDM1 was introduced into *E. coli* Xl1Blue harbouring pWSK29-M.BanLI-M.BanLII by electroporation. As expected the plasmid complement of pWSK29-M.BanLI-M.BanLII+pDM1 was resistant to restriction with AvaII, demonstrating that methylation did occur (Fig. 5). Transformation of pDM1 isolated from Xl1Blue pWSK29-M.BanLI-M.BanLII+pDM1into *B. animalis* subsp. *lactis* CNCM I-2494 gave an approximately 22-fold higher transformation efficiency as compared to unmethylated DNA (Fig. 6). This transformation efficiency is approaching that obtained with plasmid DNA isolated from *B. animalis* subsp. *lactis*.

## Discussion

It is now well established that the ingestion of functional foods or pharmabiotics containing prebiotics and/or probiotic bifidobacteria and lactobacilli can beneficially impact on the microbiota and (thereby) positively influence host physiology [2]. These beneficial effects are thought to be imparted through an array of structural and metabolic microbial features that contribute to restoring and/or maintaining gastrointestinal homeostasis, and include the ability to alter the intestinal microbiota structure or functioning, support colonization resistance against pathogens and influence host immune responses [2], [3]. The consumption of a fermented milk product containing *B. animalis* subsp. *lactis* CNCM I-2494 has been assessed in several clinical trials and found to be effective in improving digestive comfort in (healthy) adults [4], as well as in an IBS cohort study [5].

Defining how *B. animalis* subsp. *lactis* CNCM I-2494 positively contributes to host health requires an in depth molecular understanding of the probiotic attributes of this strain and this can be facilitated through comparative and functional genomics approaches coupled to host response analysis using *in vitro* or *in vivo* models. As for all bifidobacteria a major barrier to genetic manipulation of *B. animalis* subsp. *lactis* CNCM I-2494 is the lack of reliable molecular tools and procedures for members of this genetically recalcitrant genus. In addition, it is now appreciated that strain-specific R-M barriers are a contributing factor in the inability to genetically access several bifidobacterial strains. However, these R-M barriers have recently been overcome in several cases by appropriate methylation of plasmid DNA in heterologous hosts [17]–[19].

The main function of R-M systems is to protect the host against invading foreign DNA, as the unmodified incoming DNA is targeted by the restriction endonuclease component of the R-M. The host DNA is resistant to cleavage as the recognition sites of the endonuclease are modified by the cognate methyltransferase at adenosyl or cytosyl residues. *In silico* analysis of the genome of *B. animalis* subsp. *lactis* CNCM I-2494 coupled with SMRT sequencing and methylome analysis established that genomic DNA of CNCM I-2494 is methylated at two recognition sequences; the first representing a novel single stranded asymmetric adenosyl-methylation at 5′-RTC^6m^AGG-3′, and the second a cytosyl-methylation at 5′-GGW^5m^CC-3′. The latter indicates that CNCM I-2494 harbours an isoschizomer of the type IIP R-M system AvaII which was subsequently assigned to be BanLII by restriction analysis of methylated plasmid DNA. In addition, the protection afforded to genomic DNA isolated from *B. animalis* subsp. *lactis* CNCM I-2494 from restriction with commercially available AvaII was ascribed to M. BanLII. The novel single stranded adenosyl methylation at 5′-RTC^6m^AGG-3′ was attributed to M.BanLI and suggests that the BanLI R-M systems may represent an incomplete type IIS R-M system owing to the absence of a second methyltransferase, or methyltransferase domain. The presence of two methyltransferases responsible for sequence recognition and strand-specific methylation is characteristic of type IIS R-M systems, however, for *B. animalis* subsp. *lactis* CNCM I-2494 just one methyltransferase-encoding gene was present in the BanLI R-M system, and it may just be that the second methylase-encoding gene was lost due to an integration event upstream of *banLI.M*. It is thought that recognition and methylation of strand-specific sequences by type IIS R-M systems is advantageous to bacterial cells, as strands generated during DNA replication can be simultaneously protected from attack by site-specific endonucleases [54]. The prototype type IIS R-M systems is FokI, while others, such as the HgaI, MboII LlaI and LlaJI type IIS R-M systems, have been well characterized. [54]–[57]. The FokI R-M system recognizes an asymmetrical sequence and cleaves the DNA at a specific distance from the recognition sequence. M.FokI contains two functional methyltransferase domains within a single protein, each being responsible for the modification of a single DNA strand at specific adenosyl residues and while both domains are N6-adenine specific methylases, they show no significant similarity to each other outside of the sequences common to all adenine-specific methyltransferases [58]. The HgaI R-M system comprises of two cytosine methyltransferases that each methylate at a different strand. In this instance the protein sequences of the two methylases are conserved suggesting that these have evolved as a result of gene duplication [59]. Similarly, MboII, identified from *Moraxella bovis* ATCC 10900 recognizes an asymmetrical sequence, and this system comprises of two methyltransferase encoding genes *m1.mboII* and *m2.mboII*, the former specifying an adenine methyltransferase while *m2.mboII* encodes a cytosyl-methyltransferase [54]. Type IIS R-M systems have also been found to be encoded on conjugative plasmids in *Lactococcus lactis*, the LlaI R-M system was identified on a 46.2 kb plasmid, pTR2030 [60], [61], while the LlaJI R-M system was identified as one of three phage resistance systems present on the 65 kb plasmid, pNP40 [55]–[57]. The impact of the absence of the second methyltransferase for the BanLI system of *B. animalis* subsp. *lactis* CNCM I-2494 was further evident from the bacteriophage plaque assays conducted with *E. coli* DSMZ5911-pWSK29-RMBanLI where R.BanLI conferred only a very minor protection against phage infection as compared to infection of *E. coli* DSMZ5911-pWSK29 thereby suggesting that R.BanLI is not fully active. This likely represents an example of specific gene loss within the *B. animalis* subsp. *lactis* CNCM I-2494 (and apparently all known *B. animalis* subsp. *lactis* strains) genome with consequent loss of functionality of the BanLI Type IIS R-M system.

The contribution of each R-M system in impeding plasmid transformation of *B. animalis* subsp. *lactis* CNCM I-2494 was determined and it was established that both BanLI and BanLII impact on the transformation efficiency, albeit to different degrees. In agreement with our bacteriophage plaque assays and *in silico* analysis we observed only a modest 1.8 fold increase in transformation efficiency of pDM1 plasmid DNA methylated with M.BanLI despite the presence of 13 BanLI sites on this *E. coli*-bifidobacterial shuttle vector. R.BanLII was shown to present the greater obstacle to achieving a high transformation efficiency of *B. animalis* subsp. *lactis* CNCM I-2494, with pDM1. Despite just 3 BanLII sites on pDM1 a 16 fold increase in transformation efficiency of M.BanLII methylated pDM1 DNA was observed as compared to unmethyated DNA. To facilitate complete methylation of plasmid DNA by both M.BanLI and M.BanLII, and thereby enhancing the transformation efficiency of *B. animalis* subsp. *lactis* CNCM I-2494, an additional *E. coli* strain was constructed expressing both M.BanLI and M.BanLII. pDM1 isolated from this strain had a 22 fold higher transformation efficiency as compared to unmethylated DNA. Methylation of plasmid DNA with M.BanLI and M.BanLII heterologously expressed in *E. coli* allows the restriction barrier imposed by *B. animalis* subsp. *lactis* CNCM I-2494 to be overcome. The ability to overcome this restriction barrier combined with the development of molecular tools will advance research on the molecular biology, physiology and understanding of the molecular mechanisms by which *B. animalis* subsp. *lactis* CNCM I-2494 cross-talks with the human host.

## Supporting Information

Figure S1 **Comparison of the predicted banLII and banLI genetic loci of** ***B. animalis*** **subsp.** ***lactis*** **CNCM I-2494 with corresponding R-M encoding loci from other sequenced** ***B. animalis*** **subsp.** ***lactis*** **strains and other bifidobacteria.** Each solid arrow indicates an open reading frame. The lengths of the arrows are proportional to the length of the predicted open reading frame. The colour coding which is indicative of its putative function, is indicated within the arrow. Orthologs are marked with the same colour while the amino acid identity of each predicted protein is indicated as a percentage relative to its equivalent protein encoded by B. animalis subsp. lactis CNCM I-2494.
(TIF)

Figure S2 **(A) Sequence alignment of M.BanLI and M.EcoRI.** Amino acids are shaded if both of the depicted proteins contain identical residues at corresponding positions. The boxed sequences CM1 and CMII represent two of the conserved regions of N6-adenine MTases. **(B)** Alignment of the six highly conserved motifs of the predicted cytosine methyltransferase M.BanLII with annotated cytosine methyltransferases from *B. breve* UCC2003 and *B. adolescentis* ATCC 15703 and M.AluI and M.EcoRII. Highly conserved amino acids are marked with an asterix above the sequence and indicated in bold. Amino acids are shaded in grey if at least three of the depicted protein sequences contain an identical residue at a particular position.
(TIF)

Figure S3 **Plaque assays** Plaque assays were performed with *E. coli* strains DSMZ5911, DSMZ5911-pWSK29 (controls) or DSMZ5911-pWSK29- RMBanLI. The *E. coli* P1*vir* lytic phage was propagated on *E. coli* MG1655 (black bars) or *E. coli* EC100 (blue bars).
(TIF)

Table S1 **Oligonucleotide primers used in this study**
(DOCX)
