# Supplementary figures and images for: Identification of Restriction-Modification Systems of Bifidobacterium animalis subsp. lactis CNCM I-2494 by SMRT Sequencing and Associated Methylome Analysis
Source: PLoS One. 2014 Apr 17;9(4):e94875. doi: 10.1371/journal.pone.0094875 (PMC3990576; doi:10.1371/journal.pone.0094875)

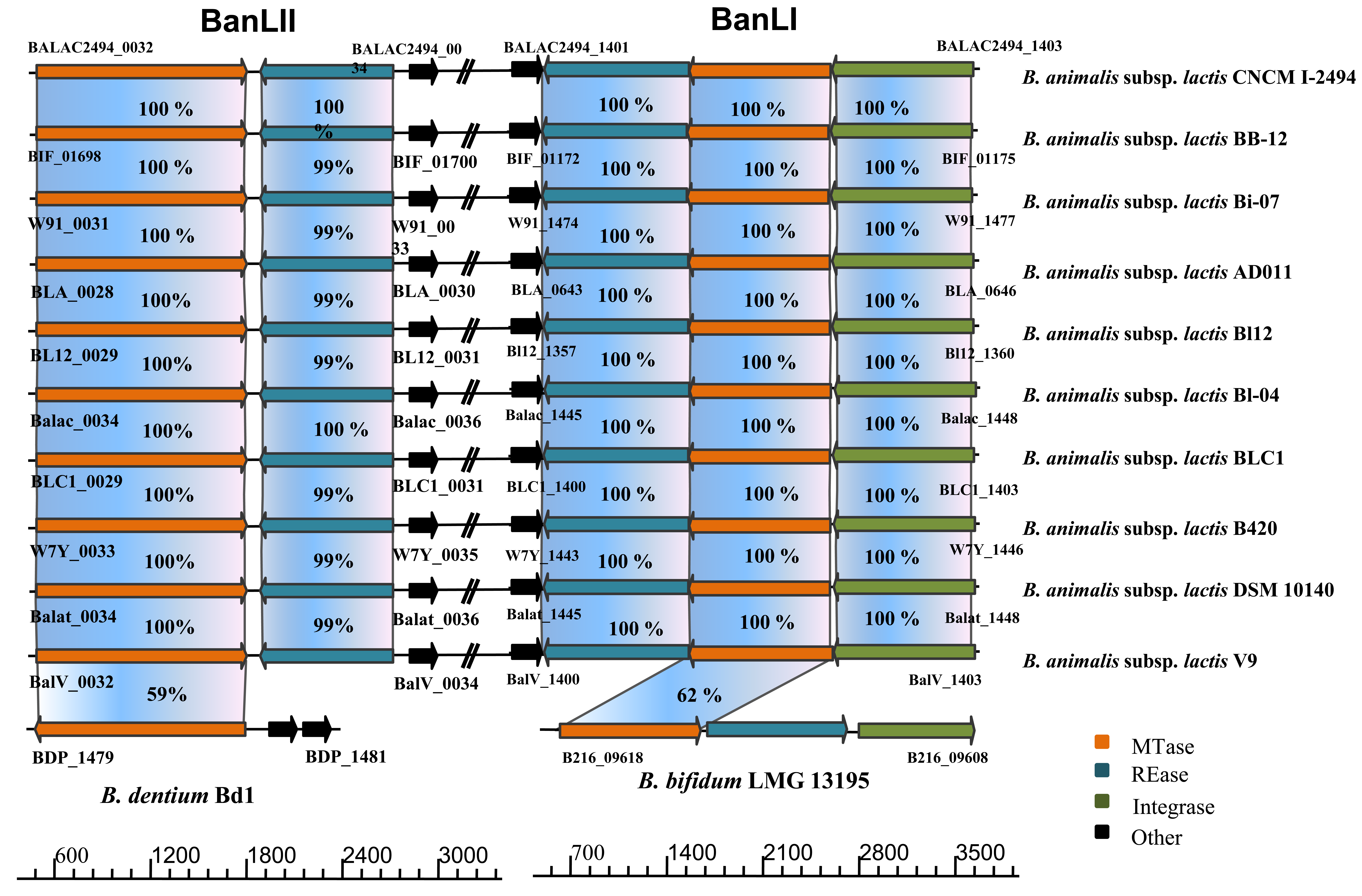

Supplement: Figure S1 — Comparison of the predicted banLII and banLI genetic loci of B. animalis subsp. lactis CNCM I-2494 with corresponding R-M encoding loci from other sequenced B. animalis subsp. lactis strains and other bifidobacteria. Each solid arrow indicates an open reading frame. The lengths of the arrows are proportional to the length of the predicted open reading frame. The colour coding which is indicative of its putative function, is indicated within the arrow. Orthologs are marked with the same colour while the amino acid identity of each predicted protein is indicated as a percentage relative to its equivalent protein encoded by B. animalis subsp. lactis CNCM I-2494. (TIF) [file pone.0094875.s001.tif]

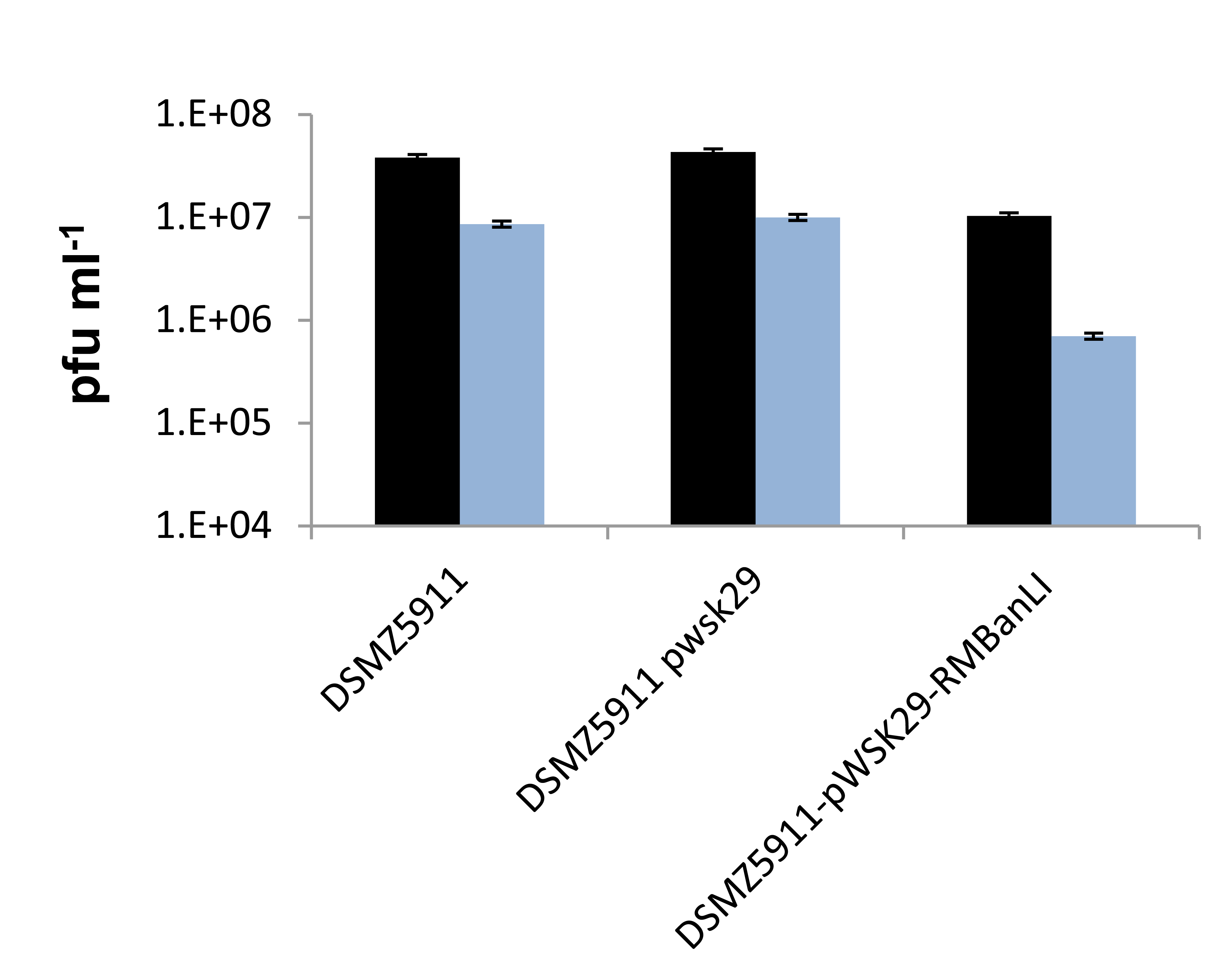

Supplement: Figure S3 — Plaque assays Plaque assays were performed with E. coli strains DSMZ5911, DSMZ5911-pWSK29 (controls) or DSMZ5911-pWSK29- RMBanLI. The E. coli P1vir lytic phage was propagated on E. coli MG1655 (black bars) or E. coli EC100 (blue bars). (TIF) [file pone.0094875.s003.tif]
