# Supplementary material for: Identification of Restriction-Modification Systems of Bifidobacterium animalis subsp. lactis CNCM I-2494 by SMRT Sequencing and Associated Methylome Analysis
Source: PLoS One. 2014 Apr 17;9(4):e94875. doi: 10.1371/journal.pone.0094875 (PMC3990576; doi:10.1371/journal.pone.0094875)
Supplement: Table S1 — Oligonucleotide primers used in this study (DOCX) [file pone.0094875.s004.docx]

**Table S1. Oligonucleotide primers used in this study**

| ***Purpose*** | ***Primer*** | ***Sequence^a^*** |
| --- | --- | --- |
| Cloning of *banL1.M.*in pNZ44 | BanLIF | cgtccg*ctgcag*ataaggaggcactcaccatggcta cgcctctcaatcgag |
|  | BanLIR | gctctat*aagctt*ttactttccttgcgcttcttc |
|  |  |  |
| Cloning of *p44-MBanLI* in pWSK29 | BanLIF1 | cgtccg*agatct*gttagttgaagaaggtttttatattacag |
| Construction of transcriptional fusion of *banLII.M* to p*lac* on pWSK29 | BanLIIF | cgtccg*tctaga*ataaggaggcactcaccatgccgcgtgtgttcaattg |
|  | BanLIIR | gctcta*ctgcag*caatggaggcgtgcaaatc |
| Construction of transcriptional fusion of RM.BanLI *to* p*lac* on pWSK29 | RM.BanLIF | cgtccg*gcggccgc*ataaggaggcactcaccatggcgaagcgagagac |
|  | RM.BanLIR | gctcta*ctgcag*cagcaaggactacgtgacag |
| Construction of pWSK29-M.BanLI-M.BanLII | BanLIF2 | tcagct*gtcgac*acaattgtaacccatacaggag |
|  | BanLIR2 | gcgacg*gtcgac*tttactttccttgcgcttcttc |
| Construction of pDM1 | SpecF | gtcctg*gagctc*gcacacgaaaaacaagttaag |
|  | SpecR | ctggaa*gagctc*caatgaataggtttacacttactttag |
| Construction of pDM2 | SpecF1 | ctggaa*aagctt*caatgaataggtttacacttactttag |
|  | SpecR1 | gtcctg*gaattc*gcacacgaaaaacaagttaag |

^a^ Restriction sites incorporated into oligonucleotide primer sequences are indicated in italix
